# Implementation of clinical audit to improve adherence to guideline-recommended therapy in acute coronary syndrome

**DOI:** 10.1186/s43044-021-00237-7

**Published:** 2022-01-12

**Authors:** Nimmy Elizabeth George, Aashiq Ahamed Shukkoor, Noel Joseph, Ramasamy Palanimuthu, Tamilarasu Kaliappan, Rajendiran Gopalan

**Affiliations:** Department of Cardiology, PSG Institute of Medical Sciences and Research, Coimbatore, India

**Keywords:** Acute coronary syndrome, Clinical audit, Evidence-based pharmacy practice, Prescriptions

## Abstract

**Background:**

Despite global consensus on the management of acute coronary syndrome (ACS), implementation of strategies to improve adherence of guideline-directed medical therapy (GDMT) remains sub-optimal, especially in developing countries. Thus, we aimed to assess the effect of clinical pharmacist-led clinical audit to improve the compliance of discharge prescriptions in patients admitted with ACS. It is a prospective clinical audit of ACS patients which was carried out for 12 months. The discharge prescriptions were audited by clinical pharmacists for the appropriateness in the usage of statins, dual antiplatelet therapy (DAPT), beta-blockers, and angiotensin-converting enzyme inhibitors (ACE-I)/angiotensin receptor blocker (ARB). A feedback report was presented every month to the cardiologists involved in the patient care, and the trend in the adherence to GDMT was analyzed over 12 months.

**Results:**

The discharge prescriptions of 1072 ACS patients were audited for the justifiable and non-justifiable omissions of mandated drugs. The first-month audit revealed unreasonable omissions of DAPT, statin, ACE-I/ARB, and beta-blockers in 1%, 0%, 14%, and 11% respectively, which reduced to nil by the end of the 11th month of the audit–feedback program. This improvement remained unchanged until the end of the 12th month.

**Conclusions:**

The study revealed that periodic clinical audit significantly improves adherence to GDMT in patients admitted with ACS.

## Background

ACS is a spectrum of clinical conditions that occur due to myocardial ischemia or infarction that are commonly due to an abrupt reduction in coronary blood flow. It comprises two clinical presentations, namely ST elevation ACS and non-ST elevation ACS [1]. The management of ACS has rapidly evolved worldwide over the past two decades with a better focus on protocol-based pharmacotherapy [2].

Modern medical treatments like percutaneous coronary intervention (PCI) are proven to have high recovery rates in patients with ACS [3]. Despite these, the survivors are still at high risk for recurrent cardiovascular events. It is estimated that the short-term mortality rate at 30 days after an acute ACS event is between 2 and 3%, whereas the rehospitalization rate within 30 days is as high as 12 to 25% [4, 5]. A significant risk persists, and the key is to reduce the morbidity and mortality risk in ACS with a secondary prevention plan [6]. Patients should inevitably receive appropriate medical management of coronary risk factors irrespective of the state of revascularization. Studies show that pharmacological strategies have improved the long-term outcome of patients presenting with ACS [7, 8].

The American College of Cardiology/American Heart Association as well as the guidelines by the European Society of Cardiology advocates the collective use of antiplatelets, angiotensin-converting enzyme inhibitors/angiotensin receptor blockers, beta-blockers, and lipid-lowering agents (primarily statins) for long-term treatment of patients after ACS [1, 9]. Many registries across the globe project that approximately one-half of patients do not receive recommended treatments after an ACS event [10]. Despite global consensus on the management of ACS, gaps in the implementation of adherence to guideline-directed therapy exist in developing countries [2]. The result from the Indian data on ACS points out that patients are less likely to receive evidence-based treatment. It also emphasizes the sub-optimal medical discharge management of ACS patients in India [11].

The purpose of the current study was to conduct a monthly clinical audit of discharge prescriptions in patients admitted with ACS. The discharge prescriptions were examined for the inclusion of recommended drugs at the recommended dosage, and the unreasonable omission of mandated drugs was highlighted to the cardiologists by clinical pharmacists. The clinical audit–feedback was carried over 1 year, and the impact of monthly clinical audits in improving the prescriptions of evidence-based pharmacotherapy in patients with ACS was analyzed.

## Methods

### Study design

A prospective, unicentric, observational clinical audit of discharge prescriptions of ACS patients was conducted at PSG Hospitals, Coimbatore, Tamil Nadu, after the clearance from the institutional human ethics committee.

### Monthly clinical audit

Adherence to GDMT is an area of prime importance in clinical medicine. Ideally, the discharge prescriptions of patients admitted with ACS should include dual antiplatelet therapy, statin, beta-blocker, and ACE-I/ARB unless contraindicated for a patient in addition to the drugs for other comorbidities. The aforementioned drugs, if missed due to valid reasons, are mentioned in the discharge summary of the patients, to avoid confusion during patient follow-up. The inclusion of the recommended drugs in ACS was audited retrospectively based on the patients’ discharge summary. This clinical audit of mandated drugs was conducted as a part of a quality improvement program in the management of ACS patients.

### Study population

The study population included all patients admitted with ACS, under the Department of Cardiology, PSG Hospitals, between June 2019 and June 2020.

### Exclusion criteria

- ACS patients discharged from hospital against medical advice
- Death

### Method

The clinical audit and feedback were conducted by cardiology clinical pharmacists. The report presentation included characteristics of patients, such as gender, age, comorbidities, type of ACS, and details about the inclusion of guideline-directed drugs in the management of ACS.

The omissions of mandated drugs were discussed upon, based on the clinical summary of the patients, and the unreasonable omissions of drugs were highlighted. This audit presentation was carried out at the end of every month to the cardiologists involved in the management of ACS patients over 12 months.

### Statistical analysis

The number of patients with unreasonable omission of DAPT, ACEI/ARB, statins, and beta-blockers were calculated in percentage. Curve estimation analysis was performed to determine the pattern of unreasonable omissions over 1 year. *p* value was considered to be significant if it was less than 0.05.

Data were analyzed using IBM SPSS statistics 24 and MS Excel (Microsoft Corp, Redmond, WA).

## Results

A total of 1072 patients were admitted with ACS in the Department of Cardiology between June 2019 and June 2020. The audit of discharge prescriptions was conducted in 1045 ACS patients who were eligible for the study. The study comprised 75.02% male patients and 24.98% female patients.

Among the ACS patients included in the study, 522 (49.9%) constituted STEMI, followed by 327 patients with NSTEMI (31.29%) and 196 patients with unstable angina (18.75%). The mean age (S.D) of the study population was 59.2 ± 12.4. The most common comorbidities among the study population were hypertension (64.6%) followed by diabetes mellitus (59.7%) and dyslipidemia (24.3%) (Table 1).

**Table 1:** Baseline characteristics of patients admitted with acute coronary syndrome

Characteristics	ACS patients (%)
Age (years)	59.2 ± 12.4
Gender	
Male	784 (75.02)
Female	261 (44.97)
Comorbidities	
Hypertension	64.6
Diabetes	59.7
Obese	1.2
Dyslipidemia	24.3
Family history of CAD	9.7
Smoking	15.8
ACS	
STEMI	522
NSTEMI	327
Unstable angina	196

### Dual antiplatelets

**Fig. 1:**
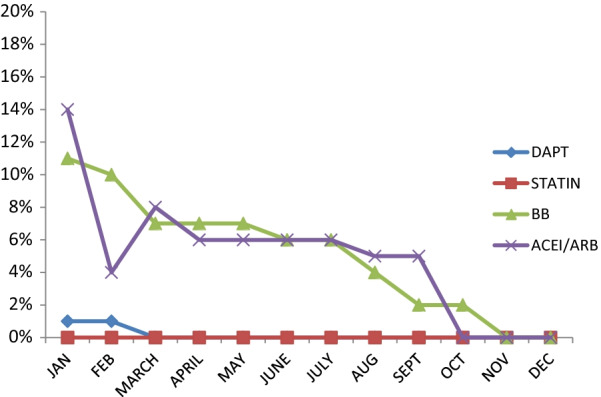
Unreasonable omission of evidence-based medications over 12 months. *ACEI* angiotensin-converting enzyme inhibitor, *ARB* angiotensin receptor blocker, *B* beta-blockers

**Fig. 2:**
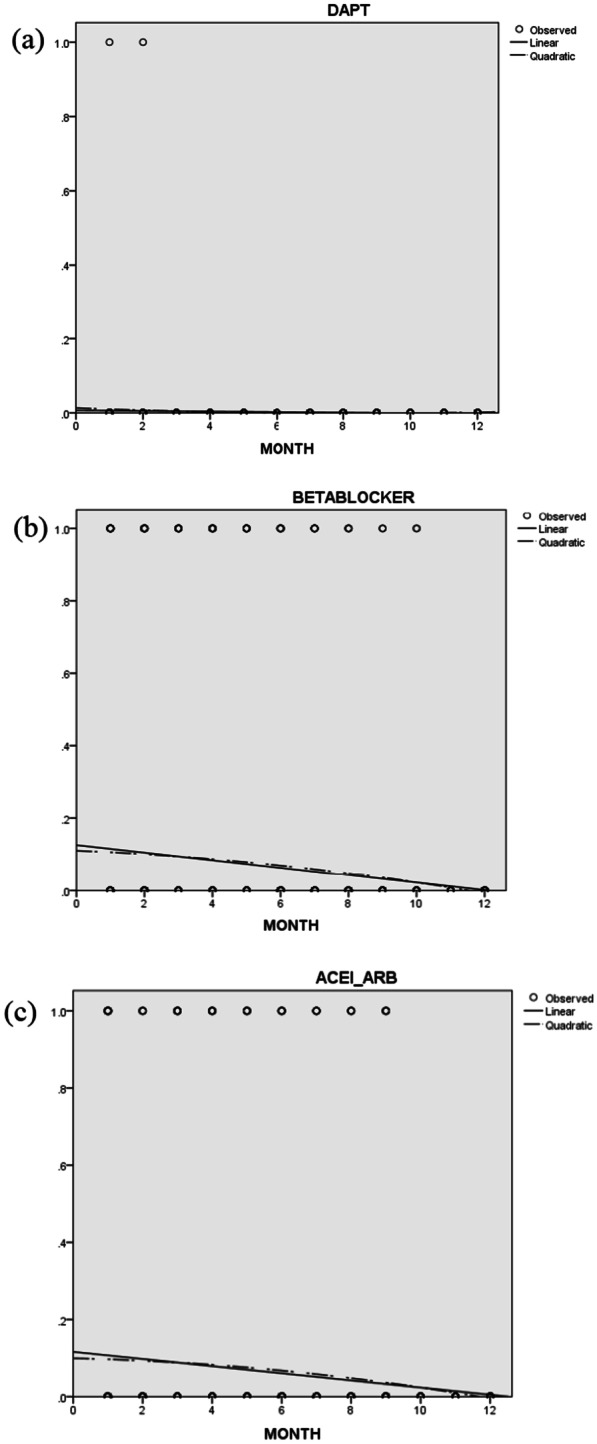
**a** Curve estimation analysis of usage of DAPT (dual antiplatelets) (linear and quadratic). **b** Curve estimation analysis of usage of *BB* beta-blockers. **c** Curve estimation analysis of usage of *ACEI* angiotensin-converting enzyme inhibitor, *ARB* angiotensin receptor blocker

The first month of audit revealed that DAPT was not included in the discharge prescriptions of 3.4% of the ACS patients. It was discussed and pointed out that 1% of patients had unreasonable omission of DAPT. The criteria to omit the drugs were agreed upon by the cardiologists, and the audit was conducted at the end of every month. The clinical audit conducted in the second month revealed a similar picture of irrational omissions of DAPT (1%). In the subsequent months, the unreasonable omissions of DAPT dropped to nil which remained unchanged throughout the study period (Table 2, Fig. 1). This was statistically significant in curve estimation analysis (Fig. 2a). Reasons for medication omission are described in Table 3.

**Table 2:** Reasonable and unreasonable omissions of drugs used during the study period

Months	Not prescribed								Total no.
	Reasonable omissions *n* (%)				Unreasonable omissions *n* (%)				
	*DAPT*	*STATIN*	*ACEI/ARB*	*BB*	*DAPT*	*STATIN*	*ACE-I/ARB*	*BB*	
1	2 (2.4)	0	1 (1.6)	7 (8.1)	1 (1.2)	0	14 (17)	11 (14)	81
2	2 (3)	0	6 (7.6)	5 (6.08)	1 (1.3)	0	4 (5.2)	10 (13)	76
3	5 (5.6)	0	9 (9.4)	10 (10.3)	0	0	8 (9)	7 (7.4)	94
4	3 (3.2)	0	4 (4.5)	10 (12.3)	0	0	6 (7.3)	7 (9)	82
5	9 (9.6)	0	8 (7.9)	8 (8.8)	0	0	6 (6.2)	7 (7.2)	96
6	4 (4.9)	0	2 (2.4)	4 (4.9)	0	0	6 (7.2)	6 (7.2)	83
7	6 (7.6)	0	5 (6.4)	4 (5.3)	0	0	6 (8)	6 (8)	76
8	9 (12.5)	1 (1.4)	5 (6.6)	4 (5.1)	0	0	5 (7)	4 (5.4)	74
9	10 (11.7)	0	6 (7.5)	6 (4.2)	0	0	5 (6)	2 (2.3)	84
10	8 (7.9)	0	6 (5.9)	8 (7.7)	0	0	0	2 (2)	99
11	11 (11.7)	0	5 (4.9)	8 (7.8)	0	0	0	0	98
12	0	4 (4)	6 (6.1)	2 (2)	0	0	0	0	102
Significance					*P* < 0.000	*P* < 0.000	*P* < 0.000	*P* < 0.000	

*ACEI* angiotensin-converting enzyme inhibitor, *ARB* angiotensin receptor blocker, *DAPT* dual antiplatelet therapy, *BB* beta-blocker, *n* number of patients

**Table 3:** Unreasonable medication omission over 12 months

Drugs	Unjustifiable reason for omission of drugs	*N*
DAPT	Medication omission error by prescriber	2
ACEI	Borderline blood pressure level	15
	Borderline potassium level	7
	Medication omission error by prescriber	11
	Transcribing error in discharge prescription	8
	Borderline serum creatinine level	6
	Contrast-induced nephropathy during hospital admission	13
Beta-blockers	Medication omission error by prescriber	19
	Non-revascularized RCA	14
	Fear of worsening lung function in patients with COPD	7
	Apprehensive to initiate beta-blocker and plan to introduce at follow-up	22

*DAPT* dual antiplatelet therapy, *ACEI* angiotensin-converting enzyme inhibitor, *RCA* right coronary artery, *COPD* chronic obstructive pulmonary disease

### Statins

The clinical audit for discharge prescriptions for ACS patients conducted in the first month revealed that all the prescriptions included statins. The audit presentation in the following months revealed 1.4% of omission in the eighth month which was justified as it was withdrawn temporarily up to short follow-up due to statin-associated muscle symptoms. The findings from successive audits showed nil omissions of statins (Table 2, Fig. 1).

### ACE-I/ARB

ACE-I/ARB was excluded in 15.6% of discharge prescriptions in the first month of observation, of which 14% of omissions were unjustified. A remarkable reduction in the omission of ACE-I/ARB (4%) was observed in the second month. A marginal rise in unreasonable omission in ACE-I/ARB was observed when compared to second month (8% in the third month and 6% in both fourth and fifth months).

The decreasing trend of unwarranted omissions of ACE-I/ARB remained the same in the sixth and seventh months as the preceding months. A decline in the unjustified omissions of ACE-I/ARB was observed in both the eighth and ninth months which showed 5% of omissions of ACE-I/ARB without genuine reason in comparison with 6% in the preceding months. The tenth-month audit was a breakthrough in terms of ACE-I/ARB omissions in which prescriptions of ACS patients with unjustified omissions were nil. This trend remained constant in the 11th and 12th months of the audit (Tables 2, 3, Figs. 1, 2c).

### Beta-blockers

The first month of audit exhibited a high number of beta-blocker omissions, which accounted for 19.1% of which only 8.1% was reasonable. The irrational omission of beta-blockers was 10% in the second month of clinical audit. A sustained decreasing trend of unreasonable omission of beta-blockers was observed in the subsequent months (7% in the third, fourth as well as fifth months). The unjustified omissions of beta-blockers in the discharge prescriptions in the sixth and seventh months of audit revealed a borderline dip (6% in both the sixth and seventh months of audit compared to 7% in the preceding months). A gradual decline in the unreasonable omissions of beta-blocker was observed in the discharge prescriptions of ACS patients in the eighth and ninth months of the audit which revealed a drop from 6% in the preceding months to 4% and 2% in the eighth and ninth months, respectively. At the end of the tenth month of audit, unwarranted exclusion of beta-blockers fell to 2%.

It was noted that, with consistent efforts, the eleventh-month audit had unreasonable omissions of all mandated drugs to zero. This trend of unreasonable omission in the key pharmacotherapy of ACS was rechecked in the 12th month of audit which once again revealed nil unreasonable omissions (Tables 2, 3, Figs. 1, 2b).

## Discussion

An optimal secondary prevention plan is indispensable in reducing CVS morbidity and mortality after an ACS event [12, 13]. Significant risks persist even after a PCI, and continuous efforts are required to reduce these risks, which can be done by optimizing pharmacological treatment at discharge and follow-up [14]. Compliance with the prescription of guideline-recommended therapy constitutes an essential quality benchmark in the management of ACS. Underutilization of GDMT is still prevalent worldwide even in developed countries [15, 16].

To the best of our knowledge, this study is the first of its kind in India, highlighting the impact of an ongoing clinical audit of discharge prescriptions in a multidisciplinary forum in improving the adherence to GDMT, resulting in optimal secondary prevention of ACS patients.

In this study, it was observed that in the first month of the audit of discharge prescriptions, only 87.1% of admitted patients were discharged collectively with all the four mandatory drugs. A study conducted in six Arab countries also revealed that only 49% of the ACS patients received evidence-based discharge prescriptions [17]. The underutilization of the evidence-based medications is observed not only in developing countries but also in many developed countries of the world. A retrospective cross-sectional study conducted in Australia and Malaysia also reported the underutilization of evidence-based pharmacotherapy in eligible ACS patients [18, 19]. Multiple ACS registries also displayed similar findings [20, 21].

The under-prescribing of essential drugs reported in previous studies is often quantified with non-adherence to GDMT. It is important to note that the exclusion of 1 or more guideline-directed pharmacotherapy in ACS does not imply non-optimal therapy. Evidence-based therapy is omitted most often due to justifiable patient-specific contraindications. This study, therefore, aims only to limit the unjustifiable omission of mandated drugs.

Clinical pharmacists are vital in the multidisciplinary management of patients with ACS [22]. A study conducted in Saudi by Amina M. Jabri et al. showed that pharmacist-led review, feedback, and discussion with treating cardiologists improved the prescription of drugs for secondary prevention in ACS from 35 to 80% [23]. Hassan et al. also reported an increase in the utilization of drugs for secondary prophylaxis with pharmacist’s involvement in clinical rounds [24]. Clinical audit has been proven to be an essential quality improvement technique [25]. Thus, we used the expertise of clinical pharmacists to conduct a clinical audit of discharge prescriptions of ACS patients in a monthly presentation in an open forum to the prescribers involved in the care of ACS patients.

Dual antiplatelet is a cornerstone of ACS management [26]. The clinical audit conducted in the first month showed 1% of the unjustifiable omission of DAPT. Presence of life-threatening bleeding, coagulopathy, thrombocytopenia, aspirin allergy, and immediate surgery such as coronary artery bypass grafting warrants an omission of DAPT. Discussion of the absolute and relative contraindication of antiplatelet drugs helped in the improvement in unjustifiable omission in discharge prescription from 1% to 0 over 12 months (*p* < 0.000) (Fig. 2a).

Contradictory to the findings of underusage of high-intensity statins in ACS in many developed countries, the retrospective audit conducted in the first month of the study period revealed that high-intensity statins were not excluded unreasonably from the discharge prescriptions of ACS patients [27]. Only the efforts to assure the existing adherence pattern had to be carried out in terms of statins, which proved successful.

It is widely known that ACE-I/ARB when initiated after an acute MI reduces mortality, recurrent CVS events, and new-onset heart failure [28]. The findings from a large US-based national registry showed that 1 in 5 eligible patients admitted with ACS failed to receive ACC/AHA class I-recommended ACE-I/ARB therapy at discharge [29]. Additionally, a study, conducted in Qatar for determining the utilization of evidence-based medication in ACS, also noted sub-optimal usage of ACE-I/ARB in comparison with other drugs [30]. In this study, it is noteworthy that 14% of eligible patients with diabetes, hypertension, chronic kidney disease, heart failure, and LV dysfunction with EF < 40% failed to receive ACE-I/ARB in the first month of observation. Current evidence suggests that physicians are ambivalent in the prescription of ACE-I/ARB probably due to concerns of worsening renal failure or hyperkalemia [31]. Consistent reinforcement of benefits and risks of these drugs with the projection of discharge prescription rates helped in the uptake of these drugs in ACS patients. At the end of 11 months, no eligible patient was discharged without an ACE-I/ARB after an ACS event (Fig. 2c).

Beta-blockers have class I indication in patients with ACS, if not contraindicated [32]. Over 11 months, the prescription rate of beta-blockers in ACS patients increased to 100%. This finding was in line with a study conducted by Hassan et al. which showed increased use of beta-blockers in cardiology units with the help of pharmacist involvement [24]. Our finding was in contrast to a study conducted by Thang Nguyen et al. which demonstrated that interventions targeted at healthcare professionals did not significantly improve the prescribing patterns in ACS except for statins [33] (Fig. 2c).

Compliance with guideline recommendations in ACS discharge management improved significantly with an ongoing audit–feedback presentation by a clinical pharmacist to the prescribing physicians.

## Conclusions

Our study provides an insight into prescription adherence to GDMT in ACS patients. It highlights the ongoing education of caregivers and reinforcement as the best practice for improving adherence to guideline recommendations. This study exhibited the striking reduction in the unjustifiable omission of dual antiplatelets, statins, ACE-I/ARB, and beta-blockers by a clinical pharmacist-led monthly audit presentation to the prescribing cardiologists. Through this study, we recommend the maintenance of GDMT checklist by clinical pharmacist before patient discharge along with clinical audit of discharge prescriptions as the best practice to improve quality of care in patients with ACS.

## Data Availability

The manuscript data are available on request to the corresponding author.

## Abbreviations

ACEI: Angiotensin-converting enzyme inhibitor
ARB: Angiotensin receptor blocker
DAPT: Dual antiplatelet
BB: Beta-blocker
*n*: Number of patients
ARNI: Angiotensin receptor neprilysin inhibitor
ACS: Acute coronary syndrome
STEMI: ST elevated myocardial infarction
NSTEMI: Non-ST elevated myocardial infarction
